# Management of Isolated Local Failures Following Stereotactic Body Radiation Therapy for Low to Intermediate Risk Prostate Cancer

**DOI:** 10.3389/fonc.2020.551491

**Published:** 2020-10-29

**Authors:** Nima Aghdam, Abigail N. Pepin, Michael Creswell, Kristin Hsieh, Clayton Smith, Nicolette Drescher, Malika Danner, Marilyn Ayoob, Thomas Yung, Siyuan Lei, Deepak Kumar, Brian Timothy Collins, Jonathan W. Lischalk, Pranay Krishnan, Simeng Suy, John Lynch, Guarav Bandi, Ryan Andrew Hankins, Sean P. Collins

**Affiliations:** ^1^Department of Radiation Medicine, Georgetown University Hospital, Washington, DC, United States; ^2^George Washington School of Medicine and Health Sciences, Washington, DC, United States; ^3^Georgetown University School of Medicine, Washington, DC, United States; ^4^Columbia University Valegos College of Physicians and Surgeons, New York, NY, United States; ^5^Department of Radiation Oncology, University of California, Los Angeles, Los Angeles, CA, United States; ^6^Geisinger Commonwealth School of Medicine, Scranton, PA, United States; ^7^Julius L. Chambers Biomedical/Biotechnology Research Institute, North Carolina Central University, Durham, NC, United States; ^8^Department of Radiology, Georgetown University Hospital, Washington, DC, United States; ^9^Department of Urology, Georgetown University Hospital, Washington, DC, United States

**Keywords:** isolated local failure, SBRT, prostate cancer, CTC, salvage therapy

## Abstract

**Background:** Stereotactic body radiation therapy (SBRT) is a safe and effective treatment option for patients with low to intermediate risk prostate cancer (1). SBRT results in very low PSA nadirs secondary to the delivery of high biologically effective doses. Studies reporting on the diagnosis, confirmation, and management of salvageable isolated local failures (ILF) are limited. This study aims to determine the incidence and management approach of ILF after SBRT in a large single institution cohort.

**Method:** All patients with low or intermediate risk localized prostate cancer treated with SBRT at Georgetown University Hospital were eligible for this study. Treatment was delivered using robotic SBRT with doses of 35–36.25 Gy in five fractions. ILF were diagnosed using multiparametric MRI and/or biopsy prompted by rising PSA levels after achieving long-term nadir. Patient's characteristics were extracted from a prospective institutional quality of life trial (IRB 2009-510). Type of salvage therapy and post-salvage PSA were determined on subsequent follow-up and chart review.

**Results:** Between December 2008 to August 2018, 998 men with low to intermediate risk prostate cancer were eligible for inclusion in this analysis. Twenty-four patients (low risk, *n* = 5; intermediate risk, *n* = 19) were found to have ILF within the prostate on either MRI (*n* = 19) and/or biopsy (*n* = 20). Median pre-treatment PSA was 7.55 ng/ml. Median time to diagnosis of ILF was 72 months (24–110 months) with median PSA at the time of ILF of 2.8 ng/ml (0.7–33 ng/ml). Median PSA doubling time was 17 months (5–47 months). Thirteen patients with biopsy proven ILF proceeded with salvage therapy (cryotherapy *n* = 12, HIFU *n* = 1). Of 12 patients who underwent cryotherapy, 7 had a post-treatment PSA of <0.1 ng/ml. One patient experienced a urethral-cutaneous fistula (grade 3 toxicity).

**Conclusion:** The incidence of isolated local recurrence is rare in our cohort. Diagnosis and management of isolated local failures post-SBRT continues to evolve. Our report highlights the importance of early utilization of MRI and confirmatory biopsy at relatively low PSA levels and long PSA doubling time (1). Additionally, undetectable PSA levels after salvage therapy supports the role of early treatment in ILF (1). Further research is needed to determine appropriate patient selection and salvage modality in this population.

## Introduction

External beam radiation therapy (EBRT) traditionally results in slow prostate specific antigen (PSA) declines that stabilize with time (2). Undetectable PSA levels following treatment are infrequent as EBRT does not fully ablate normal prostate tissue. Although EBRT achieves high early biochemical relapse free survival rates, patients may experience biochemical failure many years after undergoing treatment (3, 4). PSA rises are utilized for the early detection of recurrent disease and commonly occur years prior to clinical failure. Per the Phoenix criteria, biochemical failure is defined as at least 2 ng/mL rise in PSA above the nadir (5). Favorable prostate cancer most commonly recurs at the site of the initial tumor in the prostate bed (6–8). Local failure following definitive radiotherapy indicates a poorer prognosis (9). If left untreated, these local recurrences could lead to distant metastases and a prostate cancer-specific death (10, 11).

Brachytherapy is an ablative procedure with very low PSA nadirs (< 0.01 ng/ml) and excellent long-term outcomes with 7-year biochemical disease-free survival rates at 93–95% (12). It has recently been reported that a PSA of < 0.2 ng/ml following brachytherapy predicts for a very high probability of cure (13). One study found that individuals with a stable PSA had a median nadir of 0.03 ng/ml while those who experienced biochemical failure had a PSA median nadir of 0.5 ng/ml (14). A rising PSA after brachytherapy treatment of favorable prostate cancer by an experienced practitioner is rare.

Stereotactic body radiation therapy (SBRT) has been shown to be a safe and effective treatment option for patients with low to intermediate risk prostate cancer (15). SBRT delivers high biologically effective doses in four to five treatment sessions with 7-year biochemical disease-free survival rates in low to intermediate risk prostate cancer of 90–95% (15). PSA nadirs are lower with brachytherapy and SBRT than EBRT (16, 17). The Phoenix criteria was developed for low dose conventionally fractionated EBRT and may not be appropriate for such ablative therapies (18). Studies on the kinetics of PSA after SBRT have shown PSA rises following SBRT may reflect a benign PSA bounce or local and/or distant recurrence (16).

Strategies to optimize the accurate early identification of salvageable isolated local failures (ILF) following SBRT are needed. In general, isolated local failures occur many years post-RT (> 3 years) and are associated with long PSA doubling times (> 12 months) (19, 20). Detecting local recurrence using T2 weighted MRIs poses a challenge secondary to radiation-associated imaging changes (21). The advent of multiparametric magnetic resonance imaging (mpMRI) has enhanced the localization of prostate cancer recurrence by utilizing the combination of multiple imaging modalities (22). Previous studies suggest MRI improves localization of cancer recurrence in the setting of biochemical failure after EBRT and brachytherapy (7, 23–28).

After detection and localization of ILF after radiation therapy, multiple options for salvage therapy are available, including cryoablation and high-intensity focused ultrasound (HIFU) (29–39). Outcomes from the Cryotherapy Online Data (COLD) registry showed a 5-year actuarial biochemical disease-free rates of ~50% with focal/whole gland salvage cryoablation (40, 41). Post-cryotherapy PSA nadir < 0.5 ng/ml is the best predictor of post-cryotherapy cancer control (42). Studies on salvage therapy following SBRT are limited. Given the diagnosis and management of ILF continues to evolve in the era of successful salvage therapy, this study aims to determine the incidence and the approach to diagnosis and management of ILF after SBRT in a large single institution cohort.

## Methods

### Patient Selection and Data Collection

All individuals diagnosed with low to intermediate risk prostate cancer treated at MedStar Georgetown University Hospital between December 2008 and August 2018 were eligible for inclusion in this study. Eligible patients were required to have documented isolated local failures in the prostate and/or seminal vesicles on either mpMRI and/or TRUS biopsy following rising PSA. Patients were stratified by risk using the D'Amico classification (43). Individuals with high-risk prostate cancer were excluded from our study due to high rates of concurrent metastatic disease. Patients with nodal and/or bone metastases at time of local recurrence were also excluded. Subsequent salvage therapy and post-salvage PSA were determined by chart review. The Georgetown University Institutional Review Board (IRB) approved this single institutional retrospective review (IRB# 2009-510).

### SBRT Treatment Planning and Delivery

Simulation, contouring, and treatment planning were conducted from a previously reported institutional protocol (44). One week after placement of 4 to 6 gold fiducial markers into the prostate, patients underwent a CT and MRI for treatment planning. The clinical target volume (CTV) included the prostate and proximal seminal vesicles up to the point where the seminal vesicles separated. The planning target volume (PTV) was equal to the CTV expanded to 3 mm posteriorly and 5 mm in all other directions. The prescription isodose line was limited to >75% to restrict the maximum prostatic urethra dosage to 133% of the prescribed dose. Inverse planning was created with a prescription dose of 35–36.25 Gy in 5 fractions corresponding to an EQD2 of ~85–90 Gy assuming an alpha/beta ratio of 1.5. Treatment was delivered using CyberKnife robotic radiosurgical system (Accuray Inc, Sunnyvale, CA, USA). Given the dose inhomogeneity, it is possible to prescribe up to 40 Gy to the region of the glad with any notable lesion seen on MRI.

### Patient Follow-Up

PSA levels were obtained prior to treatment, every 3 months post-SBRT for 1 year, every 6 months for the following 2 years, and then yearly. PSA nadirs were determined and defined as the lowest PSA prior to failure. If PSA levels rose to >1 ng/ml, a digital rectal exam (DRE) was performed and a mpMRI was obtained. If there were abnormalities on the DRE and/or mpMRI, a biopsy was recommended. Following identification of ILF, patients were expectantly managed or underwent salvage therapy including HIFU and cryotherapy. Post-salvage PSA was determined. Toxicities were assessed following salvage therapy and scored using the common terminology for adverse events version 4 (CTCAE v4). This cohort predates the widespread use and availability of PSMA/Choline PET in the USA.

### Composite Representation of Localized Failures

**Figure 1:**
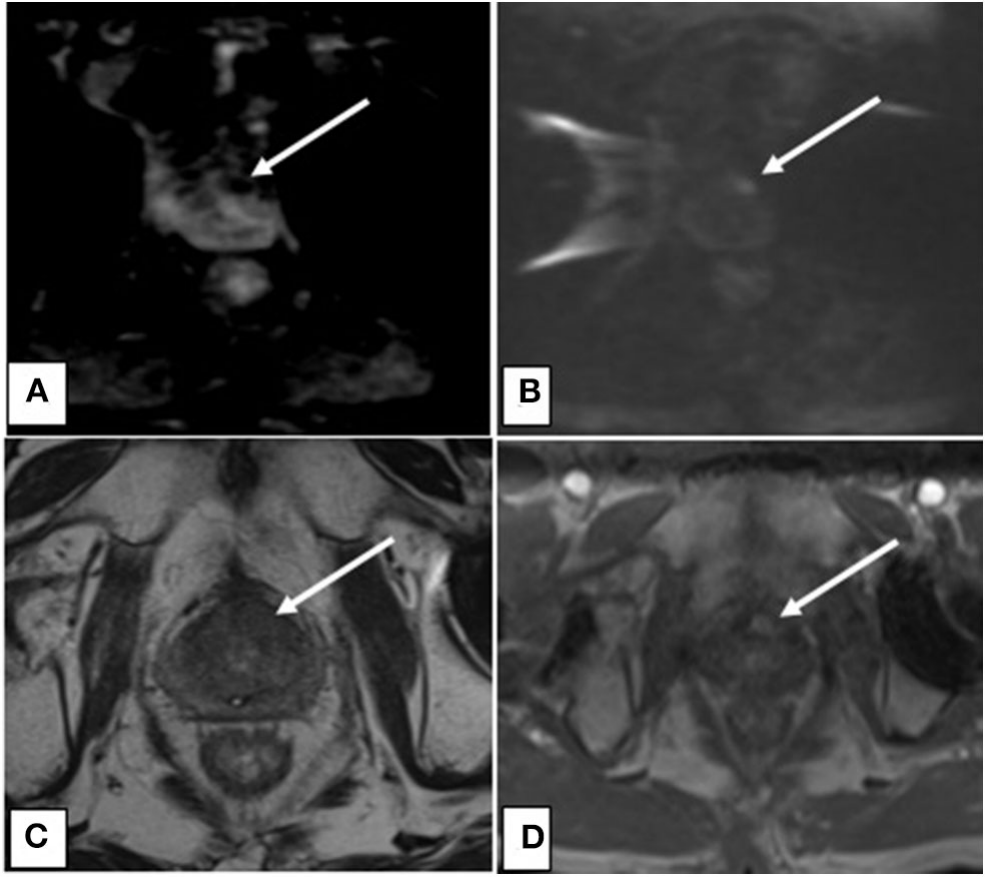
**(A)** Axial ADC map showing decreased signal intensity associated with lesion indicative of restricted diffusion. **(B)** Axial DWI showing increased signal intensity associated with lesion indicative of restricted diffusion. **(C)** Axial T2 showing decreased signal intensity associated with the lesion. **(D)** Axial DCE with contrast showing increased perfusion to the lesion.

To determine the most common locations for isolated local failures following prostate SBRT, post-SBRT imaging reports and prostate biopsies were analyzed. A sample prostate MRI at the time of ILF and the most commonly utilized sequences are represented in Figure 1. Localized failure location was coded into data fields which represented prostate sections (apex, midgland, and base) and prostatic zones (peripheral, central, transitional, and anterior fibromuscular stroma). Data collection occurred in a sequential manner. First, mpMRI reports were examined. If data fields were missing, prostate biopsy data was examined to complete the data fields. If neither mpMRI nor biopsy reports were available, data from other imaging modalities were employed. mpMRI data was sufficient in 14 of 20 patients, biopsy data was used in 5 patients, and ultrasound was used for 1 patient. The degree of involvement of a section and zone combination was determined based on the number of patients with involvement divided by the number of total patients (those with and without involvement). **Figure 3** and supplemental data.

## Results

Between December 2008 and August 2018, 998 men with low to intermediate risk prostate cancer were eligible for inclusion in this study. Isolated local failures were identified in 24 patients (2.4%) at a median follow-up of 84 months. Characteristics of the patients who experienced ILF are shown in Table 1. The median age was 66.5 (range 48–80). This patient cohort was diverse. Approximately 58% of the population was white and 33% was black. The median pre-SBRT PSA was 7.55 ng/ml (3.7–15.6 ng/ml). Approximately 21% of individuals were low-risk and 79% of individuals were intermediate risk. Neoadjuvant androgen deprivation therapy was employed in 25% of the cohort. Seventy-five percent were treated with 36.25 Gy in 5 fractions, while the remaining 25% underwent 35 Gy in 5 fractions.

**Table 1:** Patient Characteristics.

	**Percentage of patients (*n* = 24)**
Age (years)	Median 66.5 (48–80)
**Race**	
White Black Other	58.3% (14) 33.3% (8) 8.3% (2)
**Risk group (d'amico)**	
Low Intermediate	20.8% (5) 79.2% (19)
**Clinical stage**	
T1c-T2a T2b	3.3% (20) 16.7% (4)
**Gleason Score**	
6 7	29.2% (7) 70.8% (17)
PSA (ng/ml) at diagnosis	Median 7.55 (3.7–15.6)
Prostatic volume (cc) at diagnosis	Median 40.24 (17.5–90)
% total cores positive	Median 26.32 (8.33–83.33)
Maximum % of a single involved core	Median 40.24 (2–95)
**ADT**	
ADT No ADT	25% (6) 75% (18)
**Treatment dose**	
35 36.25	25% (6) 75% (18)

**Figure 2:**
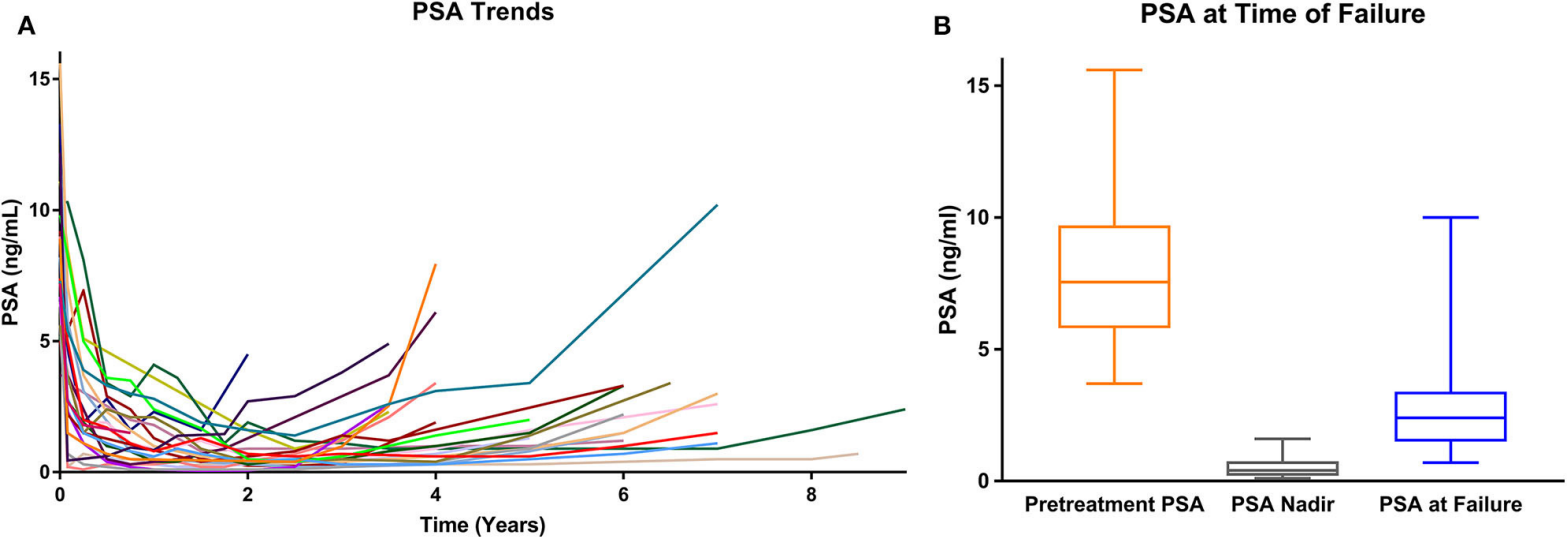
**(A)** PSA values over time for each isolated local failure. **(B)** PSA values at time of failure (whisker box plot).

In general, the PSAs declined rapidly and then rose slowly most commonly between 3 and 8 years post-SBRT (Table 2; Figure 2A). The median post-SBRT PSA nadir was 0.4 ng/ml with a median time to diagnosis of ILF was 71.8 months (23.8–110.4 months). The median PSA at failure was 2.8 ng/ml (0.7–33 ng/ml) (Figure 2B) with a median PSA doubling time of 17 months (5–47 months). Approximately 29% of the included individuals were noted to have an abnormal DRE at time of ILF. ILF was confirmed by mpMRI (79.2%) and/or TRUS biopsy (83.3%).

**Table 2:** Characteristics of Isolated Local Failure.

Median (range)	
Median time to ILF^*^ (months)	71.8 (23.8–110.4)
PSA nadir (ng/ml)	0.4 (<0.1–1.6)
PSA at failure (ng/ml)	2.8 (0.7–33)
PSA doubling time	17 (5–47)
Percentage of patients (*n* = 24)	
Failure confirmation method	
MRI Biopsy	79.2% (19) 83.3% (20)
Abnormal DRE	29.2% (7)
Salvage modality	
Cryotherapy HIFU ADT No therapy/expectant management Lost to Follow up	50% (12) 4.2% (1) 12.5% (3) 25% (6) 8.3% (2)

* *Represents actuarial median time to diagnosis of failed patients*.

**Figure 3:**
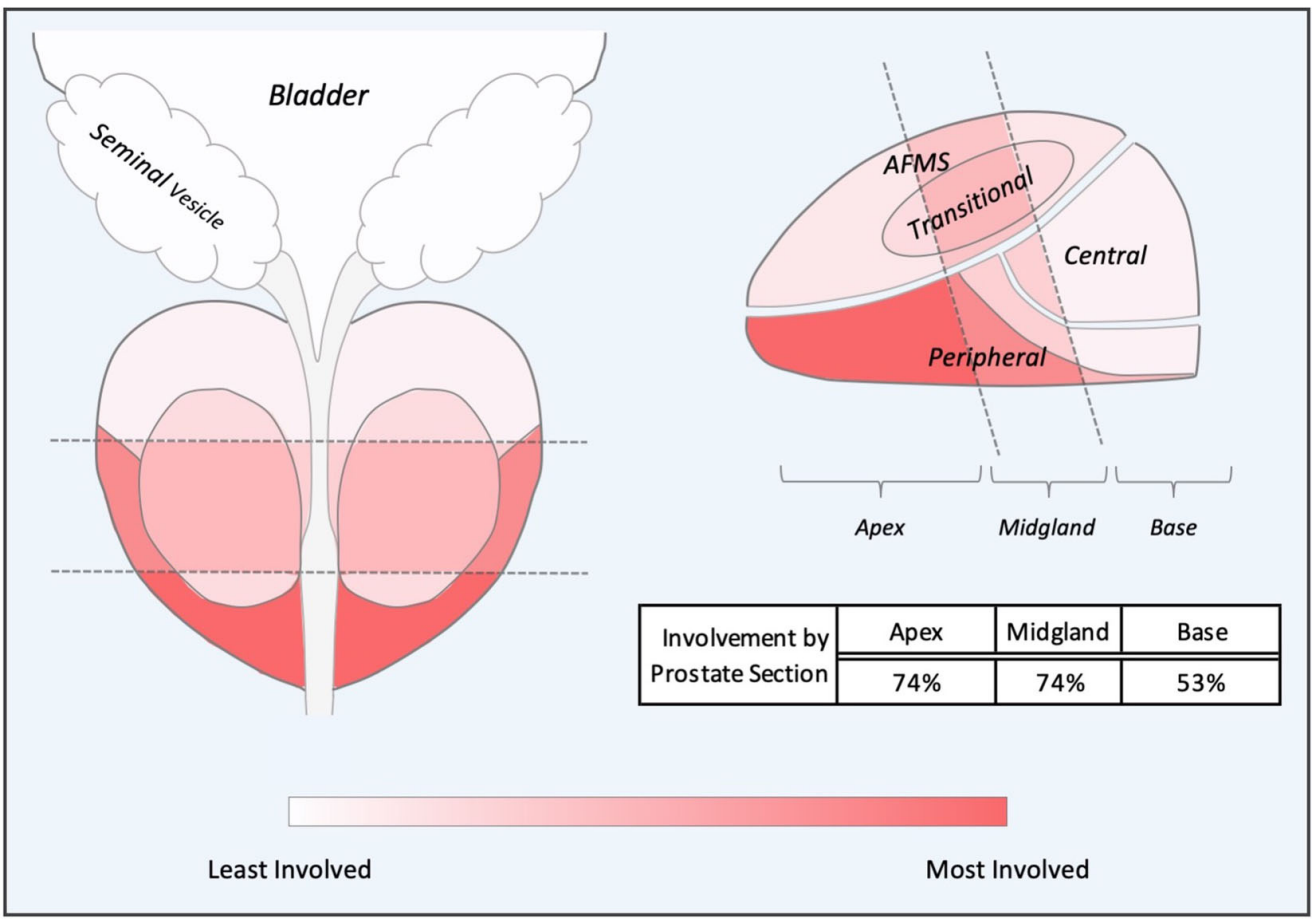
Composite Representations of Localized Failures. A depiction of the prostate, seminal vesicles and bladder shown in coronal and sagittal orientations without laterality. The prostate is subdivided into apex, midgland, and base sections and further subdivided into peripheral, transitional, central and anterior fibromuscular stroma (AFMS) prostatic zones. Sections and zones are overlaid with a color-represented heatmap representing degree of cancer recurrence involvement.

In our series, the location of mpMRI and biopsy confirmed lesions were shown to be concordant (Table 3). The sites of local recurrence documented by mpMRI and/or biopsy did not represent any one region of the prostate more than another (Table 3). The degree of involvement represented was color-coded into a heat map where the most involved areas were represented as red and the least involved were white (Figure 3). As seen previously by others, there was a trend in increased positivity from the base to the apex (45). However, there was no statistical difference in the distribution of the involved areas: apex (74%), midgland (74%) and base (53%). Of note, 25% of the patients had multifocal disease.

**Table 3:** Evaluation of individuals who experienced an isolated local failure and later underwent salvage treatment after being treated with SBRT for their prostate cancer.

**Pt**	**PSA nadir**	**Pre-salvage PSA**	**Time to Biopsy/MRI failure**	**PSA doubling time**	**Digital rectal exam**	**MRI**	**Biopsy**
1	0.8	1.4	21.7	18	Normal	Right peripheral zone	1 core involved Site: apex
2	1.4	10	58.2	22	Normal	Midline	Not performed
3	0.4	7.95	40.5	9	Normal	Left peripheral zone	1 core involved Site: left/right mid
4	0.6	1.5	57.8	18	Normal	Left side	2 cores involved Site: left anterior transition zone
5	0.3	1.1	51.1	20	Abnormal	Midline, apex	1 core involved Site: left base
6	0.4	3	37.0	17	Normal	Right peripheral zone	2 cores involved Site: right side
7	<0.1	2.6	39.9	5	Abnormal	Multifocal	12 cores involved Site: multifocal
8	0.3	6.1	42.8	10.1	Normal	Midgland to apex	7 cores involved Site: multifocal
9	0.4	1.5	47.4	17	Normal	Not performed	5 cores involved Site: unknown
10	0.1	1.5	46.8	10	Normal	Apex	3 cores involved Site: apex
11	0.2	1.9	20.4	6	Normal	Left side	6 cores involved Site: left side
12	0.2	0.7	95.1	47	Normal	Not performed	2 cores involved Site: left side
13	0.9	2.3	51.9	21	Normal	Left peripheral zone	Not performed

Thirteen patients underwent salvage therapy to treat these lesions including 92% (*n* = 12) who underwent cryotherapy and 8% (*n* = 1) who underwent HIFU (Table 2). Of the remaining individuals who chose not to proceed with salvage therapy, three individuals chose ADT, six were expectantly managed, and two were lost to follow up (Table 2). Pre-salvage PSA ranged from 0.7 to 10 ng/ml (Table 3). Post-salvage PSA ranged from <0.1–6.7 ng/ml (Table 4). Of the known post-salvage PSA readings, 58% achieved undetectable limits (Table 4). In general, whole gland salvage produced lower nadirs than focal therapy. One person experienced a urethral-cutaneous fistula (grade 3 toxicity) following whole gland cryotherapy (Table 4).

**Table 4:** Choice of salvage therapy and subsequent PSA and grade 3 toxicities in individuals experiencing an isolated failure after being treated with SBRT for their prostate cancer.

**Pt**	**Salvage technique**	**Post salvage PSA**	**Gr 3 toxicity post-salvage**
1	Whole gland cryotherapy	<0.1	Urethral-cutaneous fistula
2	Focal cryotherapy	0.7	–
3	Whole gland cryotherapy	<0.1	–
4	Focal cryotherapy	0.44	–
5	Whole gland cryotherapy	<0.1	–
6	Whole gland cryotherapy	<0.1	–
7	Whole gland cryotherapy	Unknown	–
8	Focal cryotherapy	<0.1	–
9	Whole gland cryotherapy	<0.1	–
10	Whole gland cryotherapy	<0.1	–
11	Focal cryotherapy	0.7	–
12	Focal cryotherapy	0.2	–
13	Focal HIFU	6.7	–

## Discussion

Isolated local failures were rare in this study. This is likely secondary to the high biologically effective doses (BED) administered (~200 Gy) and our inhomogeneous treatment plans which provides an intraprostatic dose of >40 Gy (44, 46). This is consistent with a recently reported Phase I dose escalation study in low to intermediate risk patients (47). The 5-year biochemical failure rates were 15, 6, 0, and 0% for 32.5 Gy, 35 Gy, 37.5 Gy, and 40 Gy, respectively (47). Unlike low dose rate brachytherapy, we did not experience any isolated bladder neck/seminal vesicle failures (48). This could be due to the exclusion of high risk patients and inclusion of the proximal seminal vesicles in the high dose volume (44). Notably, our study did not demonstrate any one area of the prostate where recurrence was occurring more frequently (6, 45). In the opinion of the authors, the recurrences identified in this study were likely secondary to innate radiation resistance rather than inadequate treatment margins (49). In the future, it will be important to determine if MRI-guided intraprostatic dose escalation can overcome innate radiation resistance and reduce local recurrences.

Similar to prior studies with alternative forms of radiation, isolated local failures were found to occur several years post-RT with long PSA doubling times (19, 20). In our population, median time to ILF was ~6 years with a median PSA doubling time of 17 months. As we continue to follow this patient cohort, we expect that isolated local recurrences will continue to occur and the median time to recurrence will increase. A recent study has suggested that a PSA nadir of < 0.2 at 4 years post treatment is the definition of cure following brachytherapy (13). In our opinions, this presumption is dangerous. At least two of our patients who were presumably cured developed isolated local failures at a later time.

The Phoenix definition for treatment failure may not be appropriate following SBRT in the era of salvage therapy. Early identification of local recurrences may increase the probability curable of salvage therapy (9). In this study, isolated recurrences were identified by MRI at a PSA of lower than 2 ng/ml in 25% of patients. Additionally, 75% of our patients achieved a post-salvage PSA nadir of < 0.5 ng/ml suggesting that we appropriately selected patients for potentially morbid salvage treatment. Morbidity was acceptable with only one patient experiencing Grade 3 toxicity.

Previous investigations have examined the use of MRI technology in the early detection of localized prostate failure. Detecting local recurrence using T2 weighted MRIs can be a challenge as they frequently show recurrences as a hyposignal in the setting of a diffuse hyposignal post-radiation prostate (21). mpMRI combines several techniques including T1-weighted, T2-weighted, diffusion-weighted imaging, dynamic contrast-enhancing imaging, and magnetic resonance spectroscopy to localize and stage recurrent prostate cancer. The FORECAST trial is currently underway assessing the accuracy of mpMRI-targeted biopsy in radiorecurrent prostate cancer (50). Our study employed a combination of mpMRI and TRUS-biopsies in the detection of isolated local failure in men treated with prostate cancer. We found mpMRI was able to detect lesions with PSA levels as low as 1.1 ng/ml.

In men with very slowly rising PSA values, it may take years for recurrences to develop into metastases and cause a patient's death (8). Many of the men with slowly rising PSA will not survive long enough to experience much of the morbidity and mortality of recurrent prostate cancer. As such, a risks vs. benefits assessment must be made as to whether the patient should undergo salvage treatment vs. expectant management. In our cohort, six men decided to proceed with observation. Mean PSA doubling time in individuals expectantly managed was 17.5 months (range 6–45.6 months). Consideration of doubling time in the context of a patient's age and comorbidities may prove critical to selecting salvage treatment vs. observation.

There are several limitations to our study. Isolated local failures were rare making it difficult to perform robust statistical analyses. In addition, most patients did not undergo fluciclovine/prostate specific membrane antigen (PSMA) PET scans at the time of first failure due to lack of availability in the United States. Additionally brachytherapy and prostatectomy, which is a viable option for patients experiencing local failure, was not offered to our patient cohort (51). This typically reflects patient preference to minimally invasive procedures. Despite this, many of these failures were likely isolated local recurrences due to low PSA nadirs following salvage treatment.

## Conclusions

The incidence of isolated local recurrence is rare in our population. Diagnosis and the management of isolated local failures after receiving SBRT for treatment of prostate cancer continues to evolve. Our findings suggest a role for the utilization of mpMRI and confirmatory biopsy in this patient population. Additionally, undetectable PSA post-salvage therapy supports early treatment of isolated local failures.

## Data Availability Statement

The datasets presented in this article are not readily available due to patient privacy concerns. Requests to access the datasets should be directed to the corresponding author.

## Ethics Statement

The studies involving human participants were reviewed and approved by Georgetown University IRB#2009-510. The patients/participants provided their written informed consent to participate in this study.

## Author Contributions

AP and NA were the lead authors, who participated in data collection, data analysis, manuscript drafting, table/figure creation, and manuscript revision. MC aided in figure creation and contributing to data collection. CS and PK aided in figure creation and manuscript drafting. KH and ND aided in data collection. MD contributed to study design and clinical data collection. SL developed the SBRT treatment plans and contributed to data analysis. JL, GB, RH, BC, and JL aided in review of the manuscript. SS is a senior author who organized the data and participated in its analysis. DK participated in data analysis and manuscript review. SC was the principal investigator who initially developed the concept of the study and the design, aided in data collected, and drafted and revised the manuscript. All authors contributed to manuscript revision, read, and approved the submitted version.

## Conflict of Interest

SC and BC serve as a clinical consultant to Accuray Inc. The remaining authors declare that the research was conducted in the absence of any commercial or financial relationships that could be construed as a potential conflict of interest.

## Abbreviations

ADT: androgen deprivation therapy
BED: biologically effective dose
CCI: Charlson Comorbidity Index
CT: computerized tomography
CTC: Common Toxicity Criteria
CTV: clinical target volume
DVH: dose-volume histogram
EBRT: external beam radiation therapy
EPIC: expanded prostate cancer index composite
GU: genitourinary
HIFU: high intensity focused ultrasound
ILF: isolated local failure
IMRT: intensity modulated radiation therapy
IPSS: international prostate symptom score
mpMRI: multiparametric MRI
MRI: magnetic resonance imaging
PET: positron emission tomography
PSA: prostate specific antigen
PSMA: prostate specific membrane antigen
PTV: planning target volume
SBRT: stereotactic body radiation therapy
TRUS: transrectal ultrasound.
